# MEN 2 syndrome masquerading as MEN 1

**DOI:** 10.1308/003588412X13171221590818

**Published:** 2012-09

**Authors:** Ezzat Tarek, Rajeev Paramesawaran, Ben Phillips, Greg Sadler

**Affiliations:** ^1^Department of Endocrine Surgery, John Radcliffe Hospital, Oxford,UK; ^2^Department of Surgery, Alexandria University,Egypt; ^3^Department of Pathology, John Radcliffe Hospital, Oxford,UK

**Keywords:** Multiple endocrine neoplasia, Medullary thyroid cancer, Cushing’s syndrome

## Abstract

Patients with multiple endocrine neoplasia (MEN) type 2A develop medullary thyroid cancer, which is associated with poor prognosis in its metastatic stage. Hyperparathyroidism is a common finding in both MEN 1 and 2. We report a 68-year-old patient diagnosed clinically with MEN 1 based on the presence of hyperparathyroidism and pituitary Cushing’s disease with no supporting genetic evidence. The hyperparathyroidism was later found to be part of MEN 2A with underlying metastatic medullary thyroid cancer. We highlight the importance of genetic confirmation before a diagnosis of MEN 1 is made as other more serious pathologies might be overlooked.

Multiple endocrine neoplasia (MEN) syndromes are rare endocrine tumour syndromes. MEN 1 syndrome involves primarily tumours of the parathyroid, pancreas and anterior pituitary gland. Primary hyperparathyroidism, the earliest and most frequent feature of MEN 1 syndrome, is also present in 20–30% of patients with MEN 2A, in addition to medullary thyroid cancer (MTC) and phaeochromocytomas.^1,2^ Patients in the general population frequently have abnormalities involving parathyroid hyperplasia and pituitary adenoma. However, endocrinologists should be careful diagnosing an MEN 1 phenotype based on these findings.

## Case history

A 68-year-old man was referred with a diagnosis of MEN 1 phenotype (negative mutation) to treat associated hyperparathyroidism. Both magnetic resonance imaging (MRI) and sestamibi scintigraphy were concordant in localising a left inferior parathyroid adenoma and the patient was listed for a parathyroidectomy.

**Figure 1:**
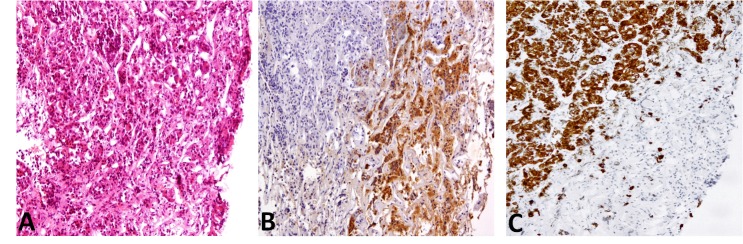
Histological sections of the pituitary gland. Unequivocal classical Cushing's adenoma is not present. The edge of one fragment is suspicious of the presence of phenotypically distinct corticotropes (A). This population of cells is positive for adrenocorticotropic hormone (B) and negative for CAM 5.2 (C).

The patient’s past medical history included diabetes and Cushing’s syndrome diagnosed in 1990. An adrenal origin to the Cushing’s syndrome was excluded due to elevated adrenocorticotropic hormone responding to a corticotropin releasing hormone test. Computed tomography (CT) showed the presence of bilateral adrenal nodules and MRI of the pituitary gland showed no evidence of a pituitary adenoma but this was confirmed with inferior petrosal sinus sampling. The patient had transphenoidal surgery to treat his Cushing’s disease both in 1992 and 2006 but this was unsuccessful. The pathology report showed an atypical appearance of a pituitary adenoma (Fig 1). Radiotherapy was considered but this was withheld until after the parathyroid surgery. He had chronic severe diarrhoea, investigated with CT of the colon and colonoscopy, which proved to be normal.

**Figure 2:**
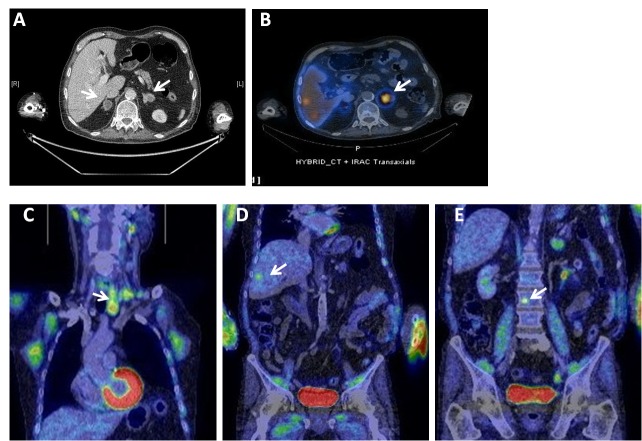
Computed tomography showed bilateral nodules (A) with the left adrenal gland showing avid uptake on the iobenguane scan in addition to liver deposits (B). Positron emission tomography showed uptake in the left lobe of the thyroid gland and cervical lymph nodes (C), segment V/VI of the liver (D) and a small deposit in the body of L3 (E).

The nodules of the adrenal glands remained stable on serial MRI and CT with negative plasma metanephrines and urinary catecholamines. Iobenguane and octreotide scans were performed to investigate the adrenal glands. The octreotide scan showed avid uptake in both adrenal glands while the iobenguane scan showed uptake in the adrenal glands with intense uptake in a left-sided thyroid nodule. Clinical examination revealed a palpable lymph node in the supraclavicular fossa. Needle cytology of the thyroid showed evidence of neuroendocrine proliferation, suggesting the possibility of MTC. Blood tests showed calcitonin levels of 27,000ng/l and carcinoembryonic antigen levels of 415μg/l. Repeated 24-hour urinary tests showed elevated catecholamines and metanephrines. Positron emission tomography showed uptake in the left thyroid lobe and cervical lymph nodes with left vocal cord palsy, in addition to uptake in both adrenal glands, the liver and spine (Fig 2).

**Figure 3:**
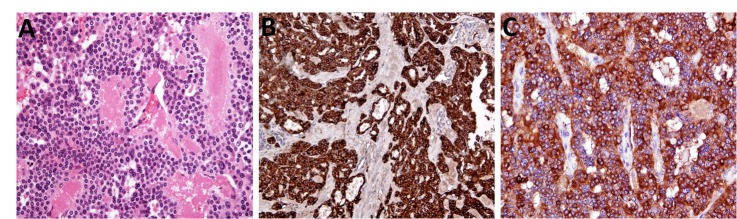
Histological sections of medullary thyroid cancer. Sections from the thyroid gland show a tumour composed of nests of cells with neuroendocrine nuclear features associated with foci of amyloid deposition (A), chromogranin (B) and calcitonin (C) positivity.

RET genetic analysis showed positive mutation of codon 634. The patient was started on phenoxybenzamine and propranolol, and listed for a bilateral adrenalectomy. However, he developed airway difficulty and a decision to perform an urgent thyroidectomy was made. A total thyroidectomy with a left neck dissection was performed. Histology of the thyroid showed MTC (Fig 3). As the patient was not symptomatic with his phaeochromocytomas, the decision was taken not to operate on the adrenal glands.

## Discussion

We report a rare case scenario of an MEN 2A syndrome overlooked by a diagnosis of MEN 1 based on phenotype with no supporting genetic abnormality. A phenotype diagnosis of MEN 1 based on parathyroid hyperplasia and pituitary adenoma alone might have serious consequences on diagnosis and management. The failure to follow the natural history of the disease might produce confusing results. This is in addition to the burden on the family members, who would require genetic testing.^3^

Our patient had incurable MTC by the time a proper diagnosis was made, with distant metastasis and locally advanced disease compromising the airway. Unexplained chronic persistent diarrhoea in the presence of hyperparathyroidism with suggestive evidence of MEN should prompt a calcitonin measurement and exclusion of MTC. More confusion was added by the asymptomatic phaeochromocytoma, which is not infrequent in 30–50% of patients with MEN 2A.^4^ In our patient, the decision to proceed with thyroid surgery despite untreated phaeochromocytomas was due to the rapid progression of the MTC with metastatic lymph node disease in the central compartment compromising the airway.

The importance of this diagnosis lies not only in managing the patient himself but in screening and treating other family members. When the patient’s son was genetically screened, he was found to carry the same genetic mutation. A prophylactic thyroidectomy was then performed showing evidence of MTC on histological examination; however, with no metastatic disease and hence associated with a more favourable prognosis.

## Conclusions

This case report highlights the importance of genetic testing in diagnosis of MEN syndromes as solely clinical diagnosis might lead to devastating consequences. Thyroid surgery for MTC in the presence of phaeochromocytoma might be attempted under certain circumstances to maintain a patent airway. Nevertheless, this needs to be performed under close monitoring with both alpha and beta receptor blockade.
